# A Novel Predictive Scoring System for 90-Day Mortality among Patients with Hepatocellular Cell Carcinoma Receiving Major Hepatectomy

**DOI:** 10.3390/cancers14061398

**Published:** 2022-03-09

**Authors:** Ruey-Shyang Soong, Yi-Chan Chen, Ta-Chun Chou, Po-Hsing Chiang, Wan-Ming Chen, Ming-Feng Chiang, Ben-Chang Shia, Szu-Yuan Wu

**Affiliations:** 1 Department of General Surgery, Chang Gung Memorial Hospital, Keelung 204, Taiwan; kodlp62@gmail.com (R.-S.S.); a017749@gmail.com (Y.-C.C.); fantasy761014@gmail.com (T.-C.C.); chiangsitoph@gmail.com (P.-H.C.); 2 Division of General Surgery, Chang Gung Medical College Taoyuan, Taoyuan 333, Taiwan; 3 Community Medicine Research Center, Chang Gung Memorial Hospital, Keelung 204, Taiwan; 4 Graduate Institute of Business Administration, College of Management, Fu Jen Catholic University, New Taipei City 242, Taiwan; c08c019@mail.pohai.org.tw (W.-M.C.); 025674@mail.fju.edu.tw (B.-C.S.); 5 Division of Gastroenterology and Hepatology, Department of Internal Medicine, Lo-Hsu Medical Foundation, Lotung Poh-Ai Hospital, Yilan 265, Taiwan; chiangmingf@gmail.com; 6 Artificial Intelligence Development Center, Fu Jen Catholic University, New Taipei City 242, Taiwan; 7 Department of Food Nutrition and Health Biotechnology, College of Medical and Health Science, Asia University, Taichung 413, Taiwan; 8 Big Data Center, Lo-Hsu Medical Foundation, Lotung Poh-Ai Hospital, Yilan 265, Taiwan; 9 Division of Radiation Oncology, Lo-Hsu Medical Foundation, Lotung Poh-Ai Hospital, Yilan 265, Taiwan; 10 Department of Healthcare Administration, College of Medical and Health Science, Asia University, Taichung 413, Taiwan; 11 Centers for Regional Anesthesia and Pain Medicine, Taipei Municipal Wan Fang Hospital, Taipei Medical University, Taipei 110, Taiwan

**Keywords:** hepatocellular carcinoma, hepatectomy, 90-day mortality, predictive scoring, overall survival

## Abstract

**Simple Summary:**

Hepatocellular carcinoma (HCC) is a major malignancy correlated with many cancer-related deaths. Surgical intervention provides superior long-term survival; however, perioperative mortality is a major concern for clinicians while making treatment decisions, especially for major hepatectomy. Scoring systems for predicting 90-day mortality in patients with HCC undergoing major hepatectomy are not available. By using the stepwise selection of the multivariate Cox proportional hazards model, we divided the patients with HCC receiving major hepatectomy into four risk groups. The Chang Gung-PohAi predictive scoring system showed significant differences in the 90-day mortality rate among the four risk groups (very low risk: 2.42%, low risk: 4.09%, intermittent risk: 17.1%, and high risk 43.6%). The Chang Gung-PohAi predictive scoring system is a promising tool for predicting 90-day perioperative mortality in patients with HCC undergoing major hepatectomy.

**Abstract:**

Purpose: Hepatocellular carcinoma (HCC) is a major malignancy and the common cause of cancer-related deaths. Surgical intervention provides superior long-term survival outcomes; however, perioperative mortality is a major concern for clinicians while making treatment decisions, especially for major hepatectomy. Scoring systems for predicting 90-day mortality in patients with HCC undergoing major hepatectomy are not available. Methods: This study used the Taiwan Cancer Registry Database that is linked to the National Health Insurance Research Database to analyze data of 60,250 patients with HCC who underwent major hepatectomy and determine risk factors to establish a novel predictive scoring system. By using the stepwise selection of the multivariate Cox proportional hazards model, we divided the patients with HCC undergoing major hepatectomy into four risk groups. Results: The Chang Gung-PohAi predictive scoring system exhibited significant differences in the 90-day mortality rate among the four risk groups (very low risk: 2.42%, low risk: 4.09%, intermittent risk: 17.1%, and high risk: 43.6%). Conclusion: The Chang Gung-PohAi predictive scoring system is a promising tool for predicting 90-day perioperative mortality in patients with HCC undergoing major hepatectomy.

## 1. Introduction

Hepatocellular carcinoma (HCC) is the leading primary malignancy of the liver [1,2]. HCC is the seventh most frequently occurring cancer globally and the second most common cause of cancer mortality [1,2]. The incidence rate of HCC is considerably high in Asia and sub-Saharan Africa [3]. Chronic hepatitis B virus (HBV) and hepatitis C virus (HCV) infections, heavy alcohol consumption, metabolic syndrome, and aflatoxin B are major risk factors for HCC [4,5]. Although the prognosis of HCC is unsatisfactory in all regions of the world, surgical intervention provides favorable outcomes in the very early (Barcelona Clinic Liver Cancer [BCLC] 0) and early (BCLC A) stages of HCC [6]. A study reported that patients beyond the early stage (BCLC stage B) could benefit from liver resection [7]. In the 1980s, liver resection resulted in a relatively high mortality rate, ranging from 10% to 30%, and was thus limited to minor resection [8]. With improvements in patient selection by using the indocyanine green (ICG) test [9], surgical techniques [10], equipment used for parenchymal transection, [11] and postoperative care, the short-term (30-day) mortality rate has substantially improved (<2%) in recent decades [12].

Surgery-related mortality is still a concern for patients and physicians while making decisions regarding the choice of curative treatment, especially major hepatectomy. A future liver remnant (FLR) volume of <26.5% in the normal liver or 31% in the cirrhotic liver after surgery can cause temporary liver failure that may trigger a cascade of complications including massive ascites, hyperbilirubinemia, coagulopathy, hepatorenal syndrome, and even mortality [13]. Technical complications of hepatectomy can result in early postoperative morbidity and mortality [8]. Patients’ outcomes after hospital discharge or within 30 days postoperatively underestimate morbidity and mortality after hepatic resection [14].

The 90-day mortality accounts for the broad spectrum of complications and deaths that can occur late in the postoperative course of patients undergoing liver resection due to systemic cascade effects exerted by deteriorating liver functions including congestive heart failure, renal failure, and sepsis [15]. Several scoring systems are regularly used by physicians to predict the perioperative 90-day mortality risk, including the American Society of Anesthesiologists (ASA) score [16,17,18] and Charlson Comorbidity Index (CCI) [19,20,21,22], especially for patients undergoing orthopedic, head and neck, and urological surgeries [23,24]. With a population of over 23 million, Taiwan has a high prevalence of HCC [25]. However, an appropriate scoring system is not yet available in Taiwan to predict the perioperative 90-day mortality of patients undergoing major hepatectomy. Therefore, we established a predictive model of 90-day mortality by using an easy-to-assess and noninvasive scoring system to rapidly evaluate the risk of perioperative mortality in patients with HCC undergoing hepatectomy.

## 2. Patients and Methods

### 2.1. Database

The study cohort was selected from the Taiwan Cancer Registry Database (TCRD, https://twcr.tw/ (accessed on 13 January 2022). We conducted a population-based cohort study by using Taiwan’s National Health Insurance (NHI) Research Database (NHIRD) linked to the TCRD. The NHIRD was established in 1979 and contains the data of 97% of cancer cases in Taiwan [26]. The NHIRD consists of all the medical claims data on disease diagnoses, procedures, drug prescriptions, demographics, and enrollment profiles of all NHI beneficiaries [27]. The NHIRD and TCRD are linked by encrypted patient identifiers. Moreover, because the NHIRD data are linked to the Death Registry, the vital status and cause of death of each patient can be ascertained. The TCRD, which is managed by the Collaboration Center of Health Information Application, contains detailed patient information on various parameters such as clinical stages, surgical procedures, techniques, and chemotherapy regimens [24,28,29,30,31,32]. 

This study was approved by the Institutional Review Board of Taipei Medical University (TMU-No. 201712019). After the scrambling of identification numbers and the deidentification of personal information, the results are made publicly available for future research purposes.

### 2.2. Selection of Study Participants

This study evaluated 60,250 patients with HCC who underwent major hepatectomy (clinical stage I–IV) between 1 January 2006, and 31 December 2017. The type of standard major hepatectomy is dependent on the location of lesions and the ability to provide an adequate FLR volume and, for malignant disease, a tumor-negative margin. Standard major hepatectomy involves the resection of two or more liver segments [33]. Patients who underwent wedge resection and one segmental resection were excluded. In addition, patients with Child–Pugh class C disease, Child–Pugh class B disease with an FLR volume of <40%, portal hypertension, and an ICG clearance of >40% at 15 min were excluded. In Taiwan, the majority of HCCs are HBV and HCV related. Usually, the HCC patients have a certain degree of fibrosis and cirrhosis. In this study, patients with portal hypertension were excluded. The diagnosis of portal hypertension based on the presence of ascites or of dilated veins or varices as seen during a physical exam of the abdomen or the anus. Various lab tests (ICG test), pre-operative computer tomography, and endoscopic exams may also be used by the professional surgeons. If the surgeon given a diagnosis of portal hypertension for the HCC patients, patients with severe portal hypertension were excluded in the study because of the high mortality of major liver resection. All of the included patients were aged >20 years. The eligible patients were divided into the following two groups based on their 90-day mortality following major hepatectomy (the index date was designated as the date on which the patients underwent major hepatectomy): 90-day mortality and 90-day survival.

### 2.3. Statistical Analysis

All statistical analyses were performed using SAS for Windows (version 9.4; SAS Institute, Cary, NC). A *p* value of ≤0.05 was considered statistically significant. Essential demographic characteristics, namely sex and age, were categorized. Patient age was determined according to the index date. Accordingly, the patients were divided into six age groups (20–29, 30–39, 40–49, 50–59, 60–69, and ≥70 years). The variables of interest were demographic characteristics; BCLC classification; American Joint Committee on Cancer (AJCC) tumor, node, and metastasis stages; presence of any other cancer, other metastatic cancers, leukemia, or lymphoma; and comorbidities. According to previous studies, comorbidities were determined from the NHIRD or TCRD [24,34,35,36]. Patients with diabetes mellitus (DM), pneumonia, chronic obstructive pulmonary disease (COPD), HBV infection, HCV infection, angina, heart valve dysfunction, sepsis, heart failure, disseminated intravascular coagulation (DIC), adult respiratory distress syndrome (ARDS), aortic aneurysm, peripheral vascular disease, peptic ulcer disease, dementia, chronic pulmonary disease, connective tissue disease, mild liver disease, moderate or severe liver disease, hemiplegia, coronary artery disease, myocardial infarction (MI), an implanted pacemaker, hypertension, chronic kidney disease (CKD), moderate or severe renal disease, end-stage renal disease, cerebral vascular accident, and transient ischemic attack were examined. Other cancer statuses and comorbid conditions reported >1 year before the index date were not included to ensure relevance. On the basis of the International Classification of Diseases, Ninth Revision, Clinical Modification diagnostic codes, comorbidities were identified if patients received a positive diagnosis in a single admission or had two or more repeated visits to outpatient departments within 1 year. The chi-square test was used to compare demographic characteristics, HCC staging, other cancer statuses, and comorbidities between the mortality and survival groups (Table 1).

All significant factors were identified to construct the Chang Gung-PohAi Major Hepatectomy Mortality Predictive Scoring System for 90-day mortality in patients with HCC undergoing major hepatectomy. Univariate and multivariate Cox proportional hazards models were used to determine the hazard ratio (HR) and 95% confidence interval (CI) of each factor (Table 2 and Table 3, respectively). The stepwise selection method was used to select all factors that significantly predicted 90-day mortality (Table 3). A forward stepwise selection method was employed to select all variables that exerted significant effects (*p* < 0.05) on the survival duration of the patients. Variables with a coefficient of >0 or an adjusted HR (aHR) of >1 were selected as risk factors to construct the Chang Gung-PohAi-Major Hepatectomy Mortality Predictive Scoring System by addition of points according to the aHR. The stepwise method modifies the forward selection technique such that effects already existing in the model do not necessarily remain there. During the stepwise selection method, duplicate entry and removal approaches were used for the forward selection and backward elimination to evaluate the contribution of effects as they were added to or removed from a model. Notably, the “minimum F-to-enter” was used to add or remove a variable. The most favorable model was chosen on the basis of the information criterion. Factors with an aHR of ≥1 were considered risk factors. The risk point for each risk factor was defined as the highest integer less than or equal to its corresponding aHRs in stepwise regression [23]. The patients were divided into four risk groups according to their risk scores (Table 4). The patients with high risk scores were predicted to have increased 90-day mortality following major hepatectomy. The long-term mortality rate determined using the Chang Gung-PohAi-Major Hepatectomy Mortality Predictive Scoring System was evaluated using the Kaplan–Meier method, and differences among the risk groups were determined using the log-rank test. A two-tailed *p* value of <0.05 was considered statistically significant.

## 3. Results

### 3.1. Demographic Characteristics of Patients with HCC Receiving Major Hepatectomy

We compared the basic data and comorbidities between the 90-day survival and 90-day mortality groups. Of the 60,250 patients enrolled in this study, 2725 (1840 [67%] men; mean [standard deviation, SD] age, 61.61 [16.43] years) died before reaching the 90-day threshold, whereas 57,525 patients (36,666 [63.7%] men; mean [SD] age, 58.39 [13.72] years) survived for more than 90 days. The 90-day mortality rate of the patients with HCC after major hepatectomy was 4.5%. The male to female ratio (63.7:36.3 vs. 67.5:32.5, *p* < 0.01), mean [SD] age (58.39 [13.72] vs. 61.61 [16.43] years, *p* < 0.01), and some comorbidities (e.g., DM, pneumonia, sepsis, heart failure, cerebral vascular disease, chronic renal disease, different stages of liver disease, HBV infection, different BCLC stages, AJCC clinical stages, and other cancer statuses) significantly differed between the 90-day survival and 90-day mortality groups (Table 1). By contrast, the proportion of the patients with HCV infection and hypertension did not significantly differ between the groups.

### 3.2. 90-Day Mortality Risk Assessment after Major Hepatectomy

We used the multivariate Cox proportional hazards model to analyze all the causes of 90-day mortality. Risk factors for 90-day mortality determined using the multivariate Cox proportional hazards model are listed in Table 2. After dividing the patients into six age groups and using the youngest age group (20–39 years) as reference, we observed no significant effect of any age group on the 90-day mortality outcome. Furthermore, no significant effect of sex on the 90-day mortality outcome was noted (aHR, 1.015; 95% CI, 0.865–1.191, *p* = 0.8537). Comorbidities, namely DM, pneumonia, COPD, heart valve dysfunction, sepsis, heart failure, DIC, peripheral vascular disease, peptic ulcer disease, dementia, chronic pulmonary disease, hemiplegia, myocardial infarction, angina, CKD, cerebral vascular, and BCLC and AJCC stages, were determined to be significant independent risk factors for 90-day mortality after major hepatectomy.

### 3.3. Stepwise Selection for 90-Day Mortality after Major Hepatectomy

Table 3 lists all significant factors determined by applying the stepwise method in the multivariate model for variable selection. On the basis of the HR, each different risk factor was assigned a score. After the stepwise selection of the multivariate Cox proportional hazards model for 90-day mortality in the patients with HCC receiving major hepatectomy, age ≥70 years (aHR: 1.462, score: 1); DM (aHR: 1.426, score: 1); pneumonia (aHR: 1.703, score: 2); sepsis (aHR: 4.762, score: 5); heart failure (aHR: 1.604, score: 2); DIC (aHR: 14.055, score: 14); peptic ulcer (aHR: 1.336, score: 1); dementia (aHR: 1.703, score: 2); myocardial infarction (aHR: 3.624, score: 4); moderate or severe renal disease (aHR: 4.477, score: 4); cerebral vascular accident (aHR: 1.48, score: 1); transient ischemic attack (aHR: 1.582, score: 2); other metastatic solid tumor (aHR: 1.421, score: 1); BCLC stage A, B, and C (aHR: 1.020, 3.557, and 4.024, respectively; score = 2, 4, and 4, respectively); and AJCC cN1 (aHR: 3.310, score: 3) were determined as significant independent risk factors for 90-day mortality after hepatectomy.

### 3.4. 90-Day Mortality Assessment Using the Chang Gung-PohAi Mortality Predictive Scoring System

The risk score (Chang Gung-PohAi cumulative score) was calculated on the basis of the accumulation of risk factors. The proportion of the patients who died within 90 days consistently increased with the accumulation of risk scores (e.g., score, 90-day mortality [0, 2.44%], [7, 11.69%], [12, 30.36%], and [18+, 65.12%]). The results of 90-day mortality assessment obtained using the Chang Gung-PohAi mortality predictive scoring system are presented in Table 4. We categorized the patients into very low risk (score = 0), low risk (score = 1–6), moderate risk (score = 7–11), and high risk (score ≥ 12) groups.

### 3.5. Kaplan–Meier Survival Curve for 90-Day Mortality Determined Using Chang Gung-PohAi, American Society of Anesthesiologists, or CCI Scores

**Figure 1:**
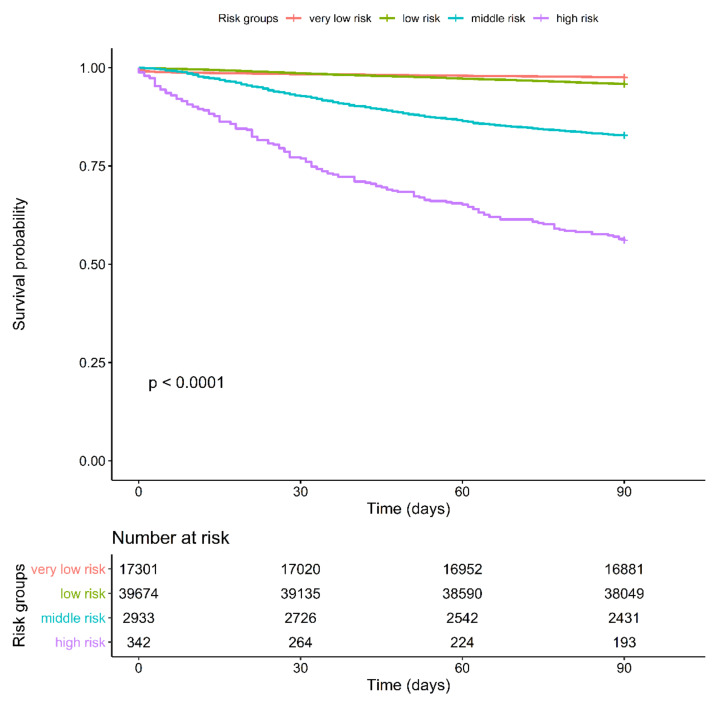
Kaplan–Meier Survival Curve for 90-Day Mortality for Four Chang Gung-PohAi-Major Hepatectomy Mortality Predictive Scoring System Groups. Note: *p* value (log-rank test) <0.0001.

As shown in Figure 1, the 90-day mortality did not significantly differ between the very-low-risk and low-risk groups. By contrast, the 90-day mortality significantly differed between the moderate-risk and high-risk groups. The American Society of Anesthesiologists (ASA) score [16] (ASA levels 1 and 2 are defined as low risk and ASA levels 3 and 4 are defined as high risk) and CCI score [19] (score: 0–5, low risk and score: 6+, high risk) are applied in some cancer surgeries to predict perioperative mortality (Tables S1 and S2). Therefore, we examined our data by using the ASA score (Figure S1) and CCI score (Figure S2). Although both the systems showed significant differences (ASA score, *P* = 0.00059; CCI score, *p* < 0.0001), the graph failed to show a distinct separation between the low-risk and high-risk groups in both the scoring systems. According to the risk score determined using the Chang Gung-PohAi predictive scoring system, the patients were divided into four groups: very low risk (score: 0), low risk (score: 1–6), intermittent risk (score: 7–11), and high risk (score: 12–18+). As shown in Figure 1, the 90-day mortality rate significantly differed among the four risk groups (very low risk: 2.42%, low risk: 4.09%, intermittent risk: 17.1%, and high risk: 43.6%). Hence, compared with the CCI and ASA scoring systems, the predictive model of 90-day mortality after hepatectomy was more favorable (Figures S1 and S2).

### 3.6. Kaplan–Meier Curve for 5-Year Overall Survival Determined Using Chang Gung-PohAi, ASA, and CCI Scores

**Figure 2:**
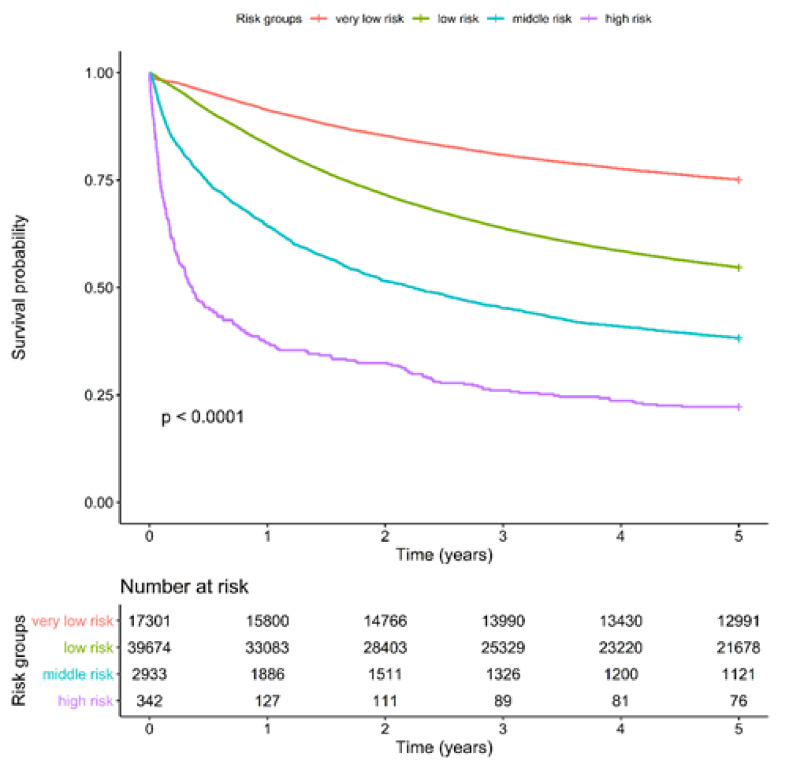
Kaplan–Meier 5-Year Survival Curve for Long-Term Mortality Rate of Four Chang Gung-PohAi-Major Hepatectomy Mortality Predictive Scoring System Groups. Note: *p* (log-rank test <0.0001).

We examined whether our scoring system can predict long-term survival. A significant 5-year survival difference was observed between the very-low-risk (75.1%) and low-risk (54.6%) groups. The other two risk groups both exhibited an early 90-day mortality trend (intermittent risk: 38.2% and high risk: 22.2%; Figure 2). The ASA scoring system exhibited a slight difference in 5-year overall survival between levels 1 and 2 and levels 3 and 4 (Figure S3). The CCI scoring system exhibited significant 5-year overall survival differences between the Gr 0–5 and Group 6+ (Figure S4).

The Chang Gung-PohAi predictive scoring system predicted not only the perioperative 90-day mortality risk but also the long-term survival outcomes of the four risk groups of the patients undergoing major hepatectomy. Furthermore, the prediction of risks and outcomes by this novel scoring system was more satisfactory than that of the ASA and CCI scoring systems for the patients with HCC undergoing receiving major hepatectomy.

## 4. Discussion

To date, an easy-to-use and a noninvasive scoring system for predicting the perioperative safety of patients undergoing major hepatectomy is not available. HCC is the leading cause of cancer deaths in China and Taiwan [37,38]. Resection of HCC with a free margin is the most vital curative treatment [39] rather than liver transplantation, especially in areas with a high prevalence of HCC [40]. However, a high 90-day mortality rate (approximately 4%) is still observed in patients undergoing major hepatectomy in Taiwan (Table 1) after preoperative evaluation performed using the Child–Pugh classification system, FLR volume examination, and ICG test at 15 min. Hence, an easy-to-use predictive tool for 90-day mortality can be valuable for patients undergoing hepatectomy in areas with a high prevalence of HCC because liver transplantation with a sufficient donor liver is impossible for most of the patients with HCC in such areas. Hepatectomy is still the major treatment modality for resecting HCC in areas with a high prevalence of HCC. Traditionally, the preoperative ICG test and liver function evaluation using Child–Pugh and liver fibrotic scores have been widely used for preoperative evaluation [41,42]. Despite the preoperative liver function evaluation, the 90-day mortality was still high in Taiwan (Table 1). Therefore, we conducted this study to establish a predictive scoring system to easily evaluate 90-day mortality that can be helpful in shared decision-making by patients with HCC and surgeons for choosing future treatment modalities. The scoring system established in this study predicted not only the 90-day mortality but also the long-term survival of the patients with HCC.

The Child–Pugh classification system has been the most widely used for assessing liver function and is crucial for predicting outcomes after hepatectomy [43]. The Fibrosis-4 (FIB-4) index is closely associated with liver fibrosis and cirrhosis [44]. The FIB-4 index effectively predicted the outcomes of patients with HCC after hepatectomy [44]. However, the FIB-4 index score predicted only the liver cirrhosis condition as the long-term outcome following hepatectomy [44]. In our study, although the prediction of the CCI scoring system was more favorable than that of the ASA scoring system, the liver fibrosis condition was not included in either of these two systems. The Chang Gung-PohAi predictive scoring system included not only the liver cirrhosis grade but also other crucial comorbidities and age. We demonstrated that by using this novel scoring system, the patients could be divided into four risk groups of 90-day mortality after major hepatectomy. Moreover, this scoring system could be used to predict the long-term survival of the patients with HCC undergoing hepatectomy.

Previously, scoring systems, such as the Child–Pugh and FIB-4 scores, based on liver function and the remaining liver volume after liver resection were used to examine mortality. In patients with less severe disease, the degree to which the underlying liver disease constitutes an absolute versus relative contraindication to hepatic resection depends upon the anticipated volume of liver remaining after resection (FLR volume) [13,45], the presence of medical comorbidities, and resources available in the event of perioperative liver failure such as the availability and proximity of liver transplantation [46]. In addition, the model for end-stage liver disease scores does not directly affect decision-making related to liver resection but may be useful in counseling a patient when choosing between liver resection and transplant [47]. For patients with normal liver function, an FLR volume of <20% increases the risk of liver failure and death following major hepatic resection [48]. Patients with mild-to-moderate underlying functional liver disease have increased risks of liver failure and death if the future liver remnant is inadequate [49]. These aforementioned evaluation tools using liver function and FLR volume have been used for a long time in Taiwan. Although all patients with HCC receiving hepatectomy in our study were evaluated using these aforementioned tools, a 90-day mortality rate of 4% was still noted in our study. The most crucial reasons might be the underlying comorbidities and age that were not considered in the evaluation.

Although FLR volume is a major factor that affects perioperative mortality in major hepatectomy, more complex systemic factors, such as chronic heart disease, cerebral vascular disease, and chronic renal function, can compete the risk of perioperative mortality [50,51]. However, no firm guidelines define what constitutes “inadequate” for specific populations receiving hepatectomy. The ASA score guide was developed by Dr. Meyer in 1941 [52] and amended in 1980. The ASA classification for a particular patient is based on systemic diseases. The extent of this disease is evident from patients’ medical history and medication list and the degree of limitation that the disease causes in patients’ everyday life. The rate of postoperative complications was found to be closely related to the ASA class (ASA score I = 0.41/1000; scores IV and V = 9.6/1000) and with emergency surgeries (ASA I = 1/1000 increases to 26.5/1000 in classes IV and V) [53]. The ASA scoring system has been widely used by clinicians in several surgical fields for determining the perioperative mortality risk. However, limited evidence indicates that the ASA score can be used to predict outcomes in patients undergoing liver resection [53]. Meanwhile, the CCI score accounts for most of the major medical comorbidities and demonstrated a reliable short-term predictive ability for postoperative patients in several surgical fields (e.g., hip surgery and urological surgery) [54]. The modified CCI score that recalibrated the weighting system more accurately predicted survival after liver transplantation [55]. However, no study has reported that the CCI score has a reliable predictive value for perioperative mortality after major hepatectomy.

The NHIRD encompasses the health information of 99% of the Taiwanese population since 1995 [56]. Research based on the NHI database provides valuable data regarding cancer diagnosis, epidemiology, outcome, and treatment [56]. The TCRD in Taiwan is a compulsory system that requires all hospitals treating patients with cancer to provide valid clinical, laboratory, imaging, pathology, and personal data to the Ministry of Health and Welfare [57]. The data were validated and generally consistent in both the databases [58]. By using accurate, large-scale, and complete data, we established the reliable Chang Gung-PohAi predictive scoring system that exhibited a significant difference in 90-day mortality among low-risk, mid-risk, and high-risk groups. By contrast, the ASA and CCI scoring systems failed to show a significant difference in survival among patients with different risks in 90 days.

There are some limitations in our study. First, based on the current findings, the clinical application of this study is limited currently because of lack of validation set. Moreover, the scoring system refers only for patients with Child Pugh A and with Child Pugh B with an FLR < 40%. However, our study included a large sample size of patients to develop a rapid assessment scoring system. Second, some comorbidities, as DIC or sepsis, have a high impact on the proposed score. However, DIC or sepsis should be effectively treated before considering a patient for major hepatectomy. It is supposed that DIC or sepsis have been be effectively treated before considering a patient for major hepatectomy. However, the patients with DIC or sepsis as the comorbidities mean the health condition or their heath environment were poorer than the patients without DIC or sepsis. Therefore, the patients with DIC or sepsis contributed to poor health conditions associated with higher mortality after major liver resection. Therefore, the predictive scoring system could give the rapid scoring to predict the 90-Day mortality after major liver resection using the easy access before surgery. Third, patients in both groups had a metastatic solid tumor and underwent hepatectomy. Patients with other metastatic solid tumor for other cancers have been under controlled in the study. For example, patients with lung adenocarcinoma with solitary brain metastasis status post ablation by stereotactic radiosurgery received hepatectomy, sequentially. It is possible for patients with a metastatic solid tumor and who underwent hepatectomy, because the ablation techniques of limited metastasis have been improving, whatever radiofrequency ablation, and microwave thermal ablation, high-intensity focused ultrasound, stereotactic radiosurgery or cryotherapy. Around 2% patients with metastatic solid tumor for other cancers receiving hepatectomy were possible, if the limited metastasis could also be removed by surgery, or other ablation therapy. Fourth, 5% of patients underwent major hepatectomy in BCLC C. If patients with BCLC stage C could pass the exclusion criteria, physicians would choose hepatectomy for them with curative-intent treatment. Therefore, 5% of patients with BCLC stage C might be possible. Because hepatectomy for patients with BCLC stage C might be a real-world clinical practice, we thought exclusion of BCLC stage C patients would be unreasonable. Consider the BCLC stage C as a part of a scoring system could be helpful to predict the 90-Day mortality after hepatectomy.

## 5. Conclusions

The novel Chang Gung-PohAi predictive scoring system, which considers comprehensive patients’ tumor-related factors, age, and systemic comorbidities, is a promising tool for predicting 90-day perioperative mortality and long-term survival in patients with HCC undergoing major hepatectomy.

## Figures and Tables

**Table 1:** Demographic Characteristics of the 90-Day Mortality and 90-Day Survival Groups of Patients with Hepatocellular Carcinoma Undergoing Major Hepatectomy.

Characteristics		90-Day Survival No. (%)	90-Day Mortality No. (%)	*p* Value
*n*		57,525 (95.5%)	2725 (4.5%)	
Sex	Female	20,859 (36.3)	885 (32.5)	<0.001
	Male	36,666 (63.7)	1840 (67.5)	
Age, mean (SD)		58.39 (13.72)	61.61 (16.43)	<0.001
Age (years)	20–29	1653 (2.9)	172 (6.3)	<0.001
	30–39	3499 (6.1)	112 (4.1)	
	40–49	8402 (14.6)	263 (9.7)	
	50–59	15,023 (26.1)	441 (16.2)	
	60–69	16,316 (28.4)	719 (26.4)	
	≥70	12,632 (22.0)	1018 (37.4)	
Comorbidities				
Diabetes mellitus	Yes	12,731 (22.1)	812 (29.8)	<0.001
Pneumonia	Yes	2859 (5.0)	320 (11.7)	<0.001
COPD	Yes	2686 (4.7)	224 (8.2)	<0.001
Hepatitis B	Yes	18,659 (32.4)	492 (18.1)	<0.001
Hepatitis C	Yes	10,065 (17.5)	447 (16.4)	0.149
Heart valve dysfunction	Yes	976 (1.7)	56 (2.1)	0.182
Sepsis	Yes	2278 (4.0)	505 (18.5)	<0.001
Heart failure	Yes	1330 (2.3)	151 (5.5)	<0.001
Disseminated intravascular coagulation	Yes	27 (0.0)	46 (1.7)	<0.001
ARDS	Yes	41 (0.1)	25 (0.9)	<0.001
Aortic aneurysm	Yes	96 (0.2)	15 (0.6)	<0.001
Peripheral vascular disease	Yes	566 (1.0)	43 (1.6)	0.003
Peptic ulcer disease	Yes	12,853 (22.3)	775 (28.4)	<0.001
Dementia	Yes	511 (0.9)	59 (2.2)	<0.001
Chronic pulmonary disease	Yes	2617 (4.5)	179 (6.6)	<0.001
Connective tissue disease	Yes	512 (0.9)	20 (0.7)	0.455
Mild liver disease	Yes	27,019 (47.0)	1100 (50.4)	<0.001
Hemiplegia	Yes	1456 (2.5)	123 (4.5)	<0.001
Moderate or severe liver disease	Yes	8362 (14.5)	413 (17.2)	<0.001
Coronal arterial disease	Yes	4390 (7.6)	278 (10.2)	<0.001
Myocardial infarction	Yes	164 (0.3)	42 (1.5)	<0.001
HTN	Yes	20,474 (35.6)	993 (36.4)	0.377
Angina	Yes	868 (1.5)	72 (2.6)	<0.001
CKD	Yes	250 (0.4)	26 (1.0)	<0.001
Moderate or severe renal disease	Yes	1622 (2.8)	425 (15.6)	<0.001
End-stage renal disease	Yes	1904 (3.3)	175 (6.4)	<0.001
Cerebral vascular accident	Yes	1738 (3.0)	165 (6.1)	<0.001
Transient ischemic attack	Yes	846 (1.5)	89 (3.3)	<0.001
Implanted pacemaker	Yes	11 (0.0)	3 (0.1)	0.016
Cancer status				
Any other cancers	Yes	14,632 (25.4)	746 (27.4)	0.025
Leukemia	Yes	44 (0.1)	3 (0.1)	0.793
Lymphoma	Yes	194 (0.3)	17 (0.6)	0.021
Metastatic solid tumor for other cancers	Yes	1229 (2.1)	72 (2.6)	<0.001
Surgical techniques				0.948
Open		54,591 (94.9)	2589 (95.0)	
Laparoscopic surgery		2934 (5.1)	136 (5.0)	
Hepatocellular cell carcinoma stages				
BCLC classification		36,768 (63.9)	1970 (72.3)	<0.001
	0	11,038 (19.2)	217 (8.0)	
	A	3312 (5.8)	134 (4.9)	
	B	3544 (6.2)	249 (9.1)	
	C	2863 (5.0)	155 (5.7)	
AJCC Clinical T stages		36,767 (63.9)	1970 (72.3)	<0.001
	cT1	11,143 (19.4)	229 (8.4)	
	cT2	3569 (6.2)	151 (5.5)	
	cT3	4496 (7.8)	273 (10.0)	
	cT4	1550 (2.7)	102 (3.7)	
Clinical N stages		36,767 (63.9)	1970 (72.3)	<0.001
	cN0	17,957 (31.2)	620 (22.8)	
	cN1	2801 (4.9)	135 (5.0)	
Clinical M stages		36,767 (63.9)	1970 (72.3)	<0.001
	cM0	18,265 (31.8)	636 (23.3)	
	cM1	2493 (4.3)	119 (4.4)	

BCLC, Barcelona Clinic Liver Cancer; COPD, chronic obstructive pulmonary disease; ARDS, adult respiratory distress syndrome; HTN, hypertension; CKD, chronic kidney disease; SD, standard deviation; T stage, Tumor stage; N stage, nodal stage; M stage, metastatic stage; AJCC, American Joint Committee on Cancer.

**Table 2:** All-Cause 90-Day Mortality Risk Assessment Using a Multivariate Cox Proportional Hazards Model in Patients with Hepatocellular Carcinoma Undergoing Major Hepatectomy.

Factor	aHR *	95% CI	*p* Value
Age (years)			
20–29	Reference		
30–39	0.745	0.302, 1.837	0.5228
40–49	0.756	0.328, 1.746	0.5129
50–59	0.857	0.379, 1.938	0.7103
60–69	1.139	0.506, 2.563	0.7536
≧70	2.117	0.944, 4.749	0.0689
Sex			
Female	Reference		
Male	1.015	0.865, 1.191	0.8537
Comorbidities			
Diabetes mellitus	1.689	1.458, 1.957	<0.001
Pneumonia	2.896	2.338, 3.588	<0.001
COPD	1.903	1.481, 2.445	<0.001
Hepatitis B	1.021	0.429, 1.585	0.4010
Hepatitis C	0.975	0.824, 1.153	0.7632
Heart valve dysfunction	1.817	1.219, 2.706	0.0033
Sepsis	8.688	7.275, 10.376	<0.001
Heart failure	2.898	2.202, 3.813	<0.001
Disseminated intravascular coagulation	35.258	15.789, 78.733	<0.001
ARDS	3.948	0.556, 28.059	0.1699
Aortic aneurysm	2.278	0.734, 7.064	0.1540
Peripheral vascular disease	2.376	1.54, 3.667	<0.001
Peptic ulcer disease	1.53	1.31, 1.786	<0.001
Dementia	2.649	1.699, 4.13	<0.001
Chronic pulmonary disease	1.353	1.004, 1.822	0.0471
Connective tissue disease	1.086	0.361, 1.799	0.5979
Mild liver disease	1.050	0.650, 1.085	0.7461
Hemiplegia	2.291	1.700, 3.087	<0.001
Moderate or severe liver disease	1.169	0.981, 1.394	0.0804
Coronal arterial disease	1.197	0.944, 1.517	0.1372
Myocardial infarction	6.275	3.824, 10.296	<0.001
HTN	1.144	0.991, 1.321	0.0661
Angina	2.058	1.445, 2.933	<0.001
CKD	2.439	1.307, 4.551	0.0051
Moderate or severe renal disease	7.185	5.98, 8.633	<0.001
End-stage renal disease	2.051	1.579, 2.664	<0.001
Cerebral vascular accident	2.251	1.718, 2.949	<0.001
Transient ischemic attack	2.516	1.792, 3.532	<0.001
Implanted pacemaker	1.000	0.999, 1.001	0.9990
Cancer status			
Any other cancers	1.029	0.858, 1.235	0.7565
Leukemia	1.000	0.991, 1.001	0.9977
Lymphoma	2.672	0.376, 18.993	0.3259
Other metastatic solid tumor	1.525	1.295, 1.797	<0.001
Surgical techniques			
Open	Reference		
Laparoscopic surgery	0.996	0.782, 3.124	0.8697
Hepatocellular cell carcinoma stages			
BCLC classification 0	Reference		
BCLC classification A	2.043	1.647, 2.534	<0.001
BCLC classification B	3.490	2.908, 4.187	<0.001
BCLC classification C	2.705	2.201, 3.325	<0.001
AJCC cT1	Reference		
AJCC cT2	2.042	1.663, 2.508	<0.001
AJCC cT3	2.898	2.431, 3.455	<0.001
AJCC cT4	3.126	2.475, 3.948	<0.001
AJCC cN0	Reference		
AJCC cN1	3.015	1.471, 4.579	<0.001
AJCC cM0	Reference		
AJCC cM1	1.361	1.119, 1.655	0.002

BCLC, Barcelona Clinic Liver Cancer; COPD, chronic obstructive pulmonary disease; ARDS, adult respiratory distress syndrome; HTN, hypertension; CKD, chronic kidney disease; SD, standard deviation; T stage, tumor stage; N stage, nodal stage; M stage, metastatic stage; AJCC, American Joint Committee on Cancer; HR, hazard ratio; CI, confidence interval. * All variables were used in multivariate analysis.

**Table 3:** Stepwise Selection of the Multivariate Cox Proportional Hazards Model for All-Cause 90-Day Mortality in Patients with Hepatocellular Carcinoma Undergoing Major Hepatectomy.

Factor	HR *	Score
Age: 30–39 years	0.854	0
Age: 40–49 years	0.974	0
Age: 50–59 years	0.817	0
Age: 60–69 years	0.967	0
Age: ≧70 years	1.462	1
Comorbidities		
Diabetes mellitus	1.426	1
Pneumonia	1.703	2
Hepatitis B	0.910	0
Sepsis	4.762	5
Heart failure	1.604	2
Disseminated intravascular coagulation	14.055	14
Peptic ulcer disease	1.336	1
Dementia	1.703	2
Myocardial infarction	3.624	4
HTN	0.998	0
Moderate or severe renal disease	4.477	4
Cerebral vascular accident	1.48	1
Transient ischemic attack	1.582	2
Cancer status		
Any other cancers	0.942	0
Other metastatic solid tumor	1.421	1
Hepatocellular cell carcinoma status		
BCLC classification A	1.929	2
BCLC classification B	3.557	4
BCLC classification C	4.024	4
AJCC cN1	3.272	3

BCLC, Barcelona Clinic Liver Cancer; COPD, chronic obstructive pulmonary disease; ARDS, adult respiratory distress syndrome; HTN, hypertension; CKD, chronic kidney disease; SD, standard deviation; T stage, tumor stage; N stage, nodal stage; M stage, metastatic stage; AJCC, American Joint Committee on Cancer; HR, hazard ratio. * All variables were used in multivariate analysis.

**Table 4:** All-Cause 90-Day Mortality Assessment Using Taiwan-Major Hepatectomy Mortality Predictive Scoring System for Hepatocellular Carcinoma.

Chang Gung-Poh Ai Cumulative Score	Survivors	Deaths	90-Day Mortality Rate after Major Hepatectomy
0	17,301	422	2.44%
1	16,552	445	2.69%
2	8651	337	3.90%
3	4067	207	5.09%
4	3398	186	5.47%
5	4287	250	5.83%
6	2719	224	8.24%
7	1257	147	11.69%
8	676	100	14.79%
9	438	94	21.46%
10	344	103	29.94%
11	218	60	27.52%
12	112	34	30.36%
13	71	23	32.39%
14	61	32	52.46%
15	25	13	52.00%
16	24	17	70.83%
17	6	3	50.00%
18+	43	28	65.12%

## Data Availability

The data sets supporting the study conclusions are included in this manuscript and its Supplementary Files.

## Supplementary Materials

The following supporting information can be downloaded at: https://www.mdpi.com/article/10.3390/cancers14061398/s1, Figure S1: Kaplan–Meier Survival Curve for 90-Day Mortality for Two Charlson Comorbidity Index score groups; Figure S2: Kaplan–Meier Survival Curve for 90-Day Mortality for Two Charlson Comorbidity Index Score Groups; Figure S3: Kaplan–Meier 5-Year Survival Curve for Long-Term Mortality Rate of Four American Society of Anesthesiologists Physical Status Classification Groups; Figure S4: Kaplan–Meier 5-Year Survival Curve for Long-Term Mortality Rate of Two Charlson Comorbidity Index Score Groups; Table S1: All-Cause 90-Day Mortality Assessment Using the American Society of Anesthesiologists Physical Status Classification Among Patients with Hepatocellular Carcinoma Undergoing Major Hepatectomy; Table S2: All-Cause 90-Day Mortality Assessment Using the Charlson Comorbidity Index Scores Among Patients with Hepatocellular Carcinoma Undergoing Major Hepatectomy.

## Abbreviations

HCC: Hepatocellular carcinoma; HBV, hepatitis B virus; HCV, hepatitis C virus; BCLC, Barcelona Clinic Liver Cancer; ICG, indocyanine green; FRL, future remnant liver; ASA, American Society of Anesthesiologists; CCI, Charlson Comorbidity Index; TCRD, Taiwan Cancer Registry Database; NHI, National Health Insurance; NHIRD, National Health Insurance Research Data; DM, diabetes mellitus; COPD, chronic obstructive pulmonary disease; DIC, disseminated intravascular coagulation; ARDS, adult respiratory distress syndrome; CAD, coronal arterial disease; MI, myocardial infarction; CKD, chronic kidney disease; ESRD, End-stage renal disease; CVA, cerebral vascular accident; TIA, transient ischemic attack; HR, hazard ratio; CI, confidence interval; SD, standard deviation; FIB-4, fibrosis index; AJCC, American Joint Committee on Cancer.
