# Supplementary material for: A Novel Predictive Scoring System for 90-Day Mortality among Patients with Hepatocellular Cell Carcinoma Receiving Major Hepatectomy
Source: Cancers (Basel). 2022 Mar 9;14(6):1398. doi: 10.3390/cancers14061398 (PMC8945917; doi:10.3390/cancers14061398)
Supplement: Supplementary file 1 [file cancers-14-01398-s001.zip › cancers-1616317-supplementary.pdf]

Table S1. All-Cause 90-Day Mortality Assessment Using the American Society of Anesthesiologists Physical Status Classification Among Patients with Hepatocellular Carcinoma Undergoing Major Hepatectomy.

ASA Cumulative Scores	Major Hepatectomy Survivors	Deaths	90-Day Mortality Rate after Major Hepatectomy
1	3199	195	6.10%
2	20343	783	3.85%
3	29414	893	3.04%
4	7294	854	11.71%

Table S2. All-Cause 90-Day Mortality Assessment Using the Charlson Comorbidity Index Scores Among Patients with Hepatocellular Carcinoma Undergoing Major Hepatectomy.

CCI Cumulative Scores	Major Hepatectomy Survivors	Deaths	90-Day Mortality Rate after Major Hepatectomy
0	4812	258	5.36%
1	1829	25	1.37%
2	9156	276	3.01%
3	5571	217	3.90%
4	3595	166	4.62%
5	8061	270	3.35%
6	8464	300	3.54%
7	3150	207	6.57%
8	9598	454	4.73%
9	1996	183	9.17%
10	1697	134	7.90%
11	1108	99	8.94%
12	649	57	8.78%
13	326	40	12.27%
14	168	28	16.67%
15+	70	11	15.71%

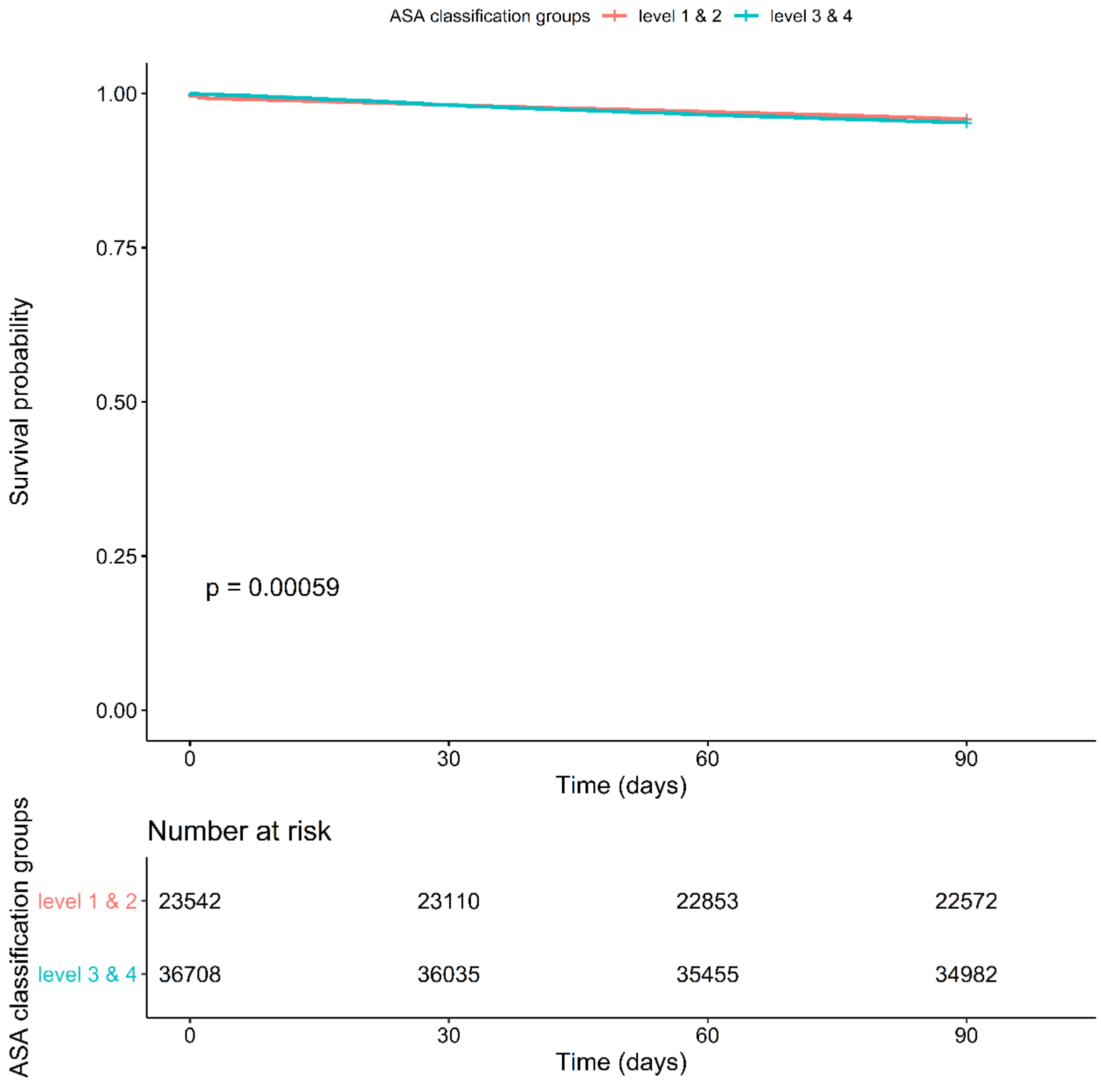

Figure S1. Kaplan–Meier Survival Curve for 90-Day Mortality for Four American Society of Anesthesiologists Physical Status Classification Groups. Note: *P* (log-rank test) = 0.00059.

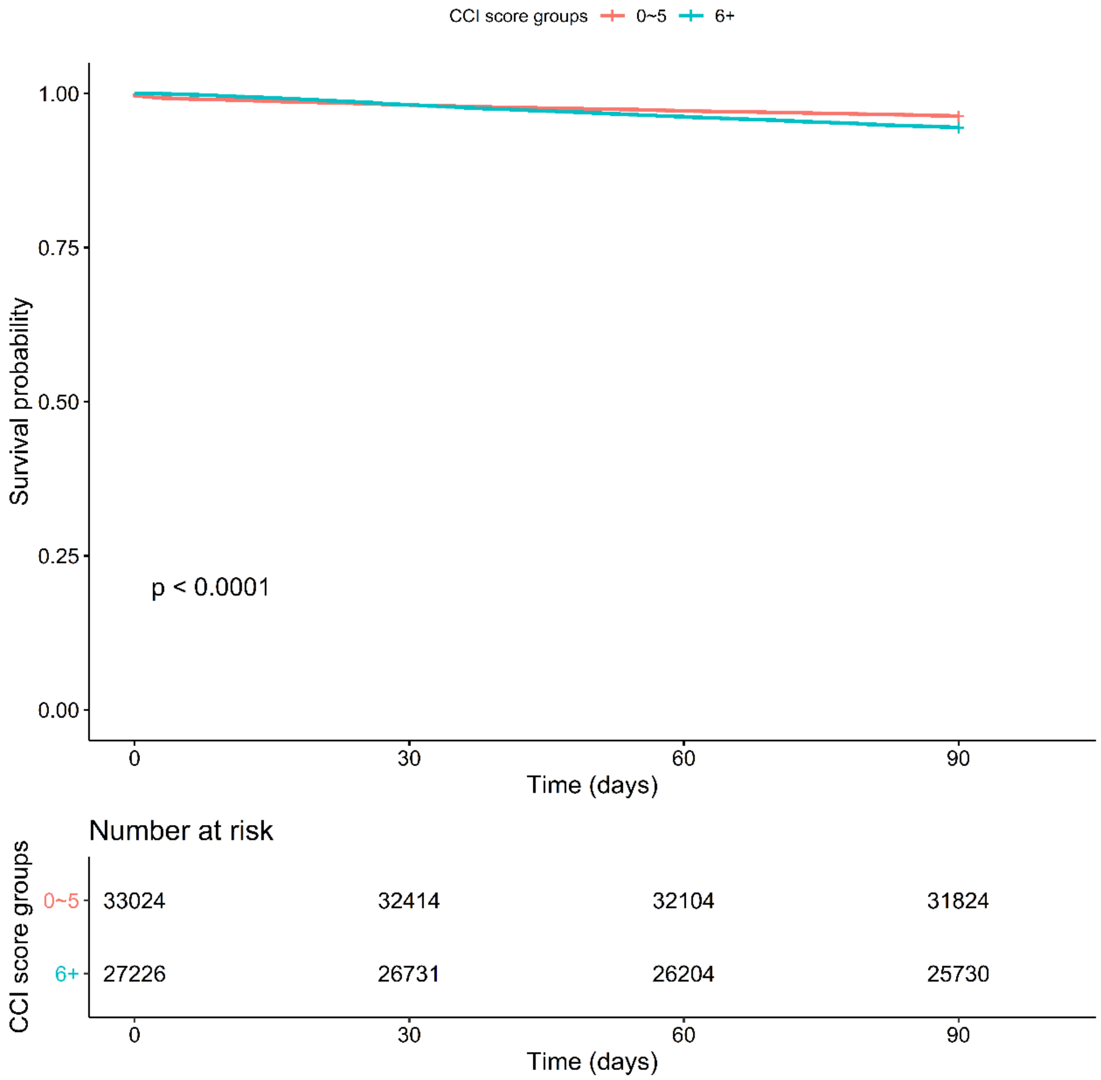

Figure S2. Kaplan–Meier Survival Curve for 90-Day Mortality for Two Charlson Comorbidity Index Score Groups. Note: *P* (log-rank test) < 0.0001.

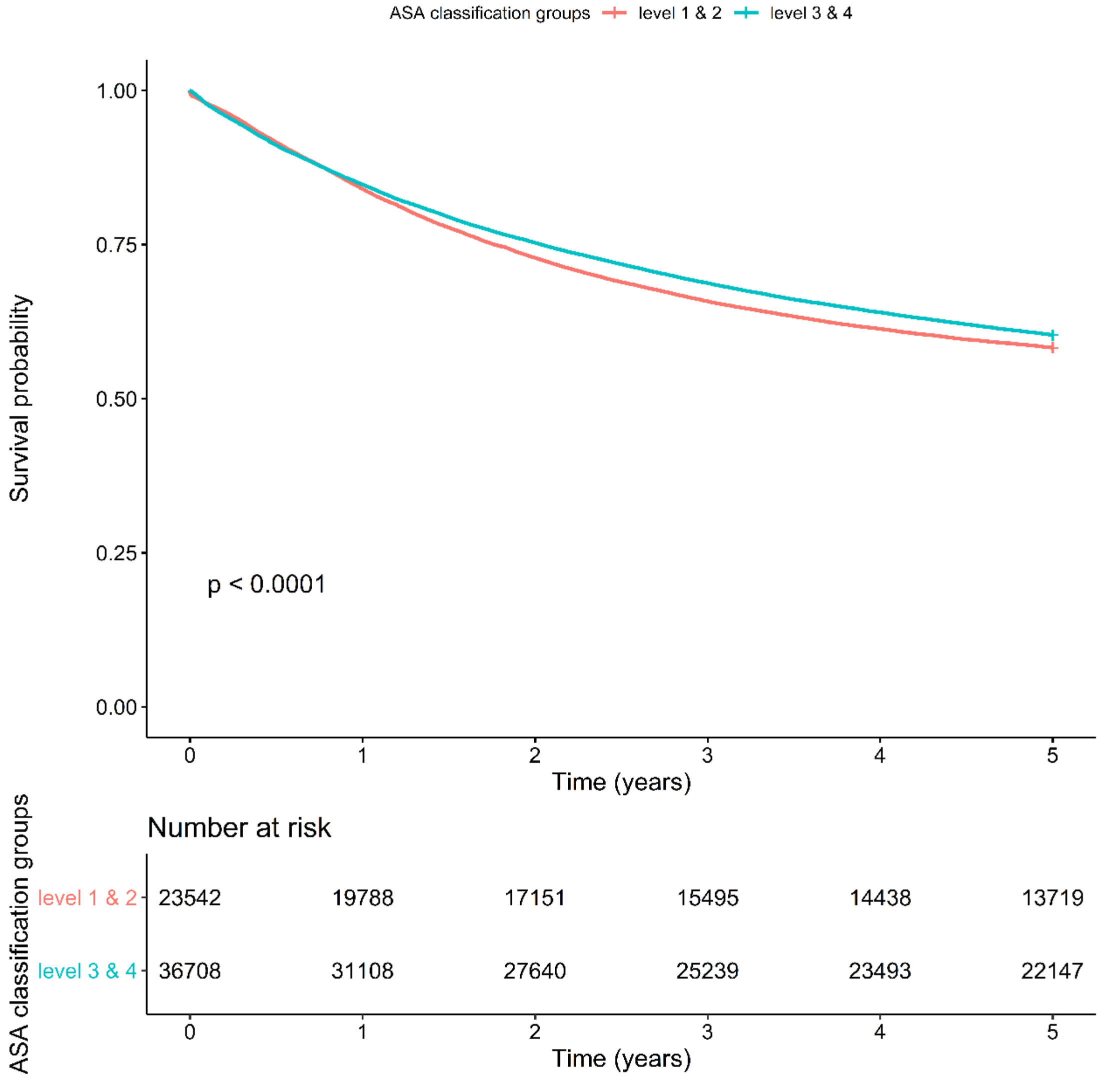

Figure S3. Kaplan–Meier 5-Year Survival Curve for Long-Term Mortality Rate of Four American Society of Anesthesiologists Physical Status Classification groups. Note: *P* (log-rank test) < 0.0001.

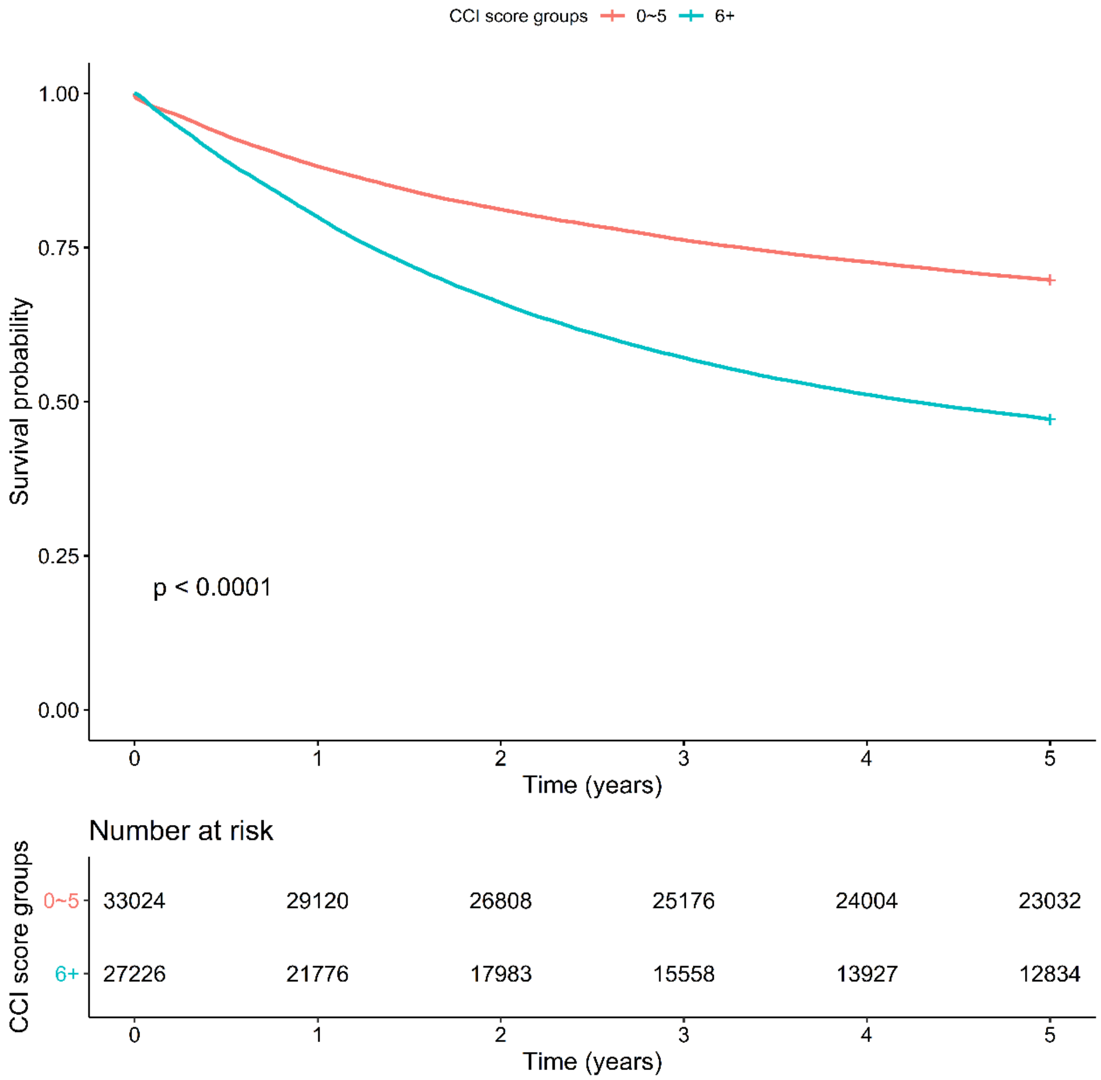

Figure S4. Kaplan–Meier 5-Year Survival Curve for Long-Term Mortality Rate of Two Charlson Comorbidity Index Score Groups. Note: *P* (log-rank test) < 0.0001.
